# Supplementary material for: Deletion of the deISGylating enzyme USP18 enhances tumour cell antigenicity and radiosensitivity
Source: Br J Cancer. 2020 Nov 20;124(4):817–30. doi: 10.1038/s41416-020-01167-y (PMC7884788; doi:10.1038/s41416-020-01167-y)
Supplement: Supplementary file 1 — Supplementary Table 1 [file 41416_2020_1167_MOESM1_ESM.docx]

| **Antibody** | **Application** | **Brand** | **Cat. No.** |
| --- | --- | --- | --- |
| USP18 | Immunoblot / Immunofluorescence | Cell Signaling -CST | 4813 |
| ISG15 | Immunoblot / Immunofluorescence | CST | 2743 |
| ISG15 | Immunoprecipitation | Boston Biochem | A-830 |
| Ubiquitin (Ubisite) | Immunoblot | Millipore | MAB5486 |
| NEDD8 | Immunoblot | CST | 2745 |
| dsRNA | Immunofluorescence | Millipore | MABE1134 |
| ADAR | Immunoblot | CST | 14175 |
| phospho STAT1 (Y701) | Immunofluorescence | CST | 9167 |
| HERC5 | Immunoblot | Invitrogen | 703675 |
| IFIT3 | Immunoblot | Abcam | ab76818 |
| PKR | Immunoblot | CST | 12297 |
| phospho PKR (T446) | Immunoblot | Abcam | ab32036 |
| IFNγ | ELISA | BD | 555142 |
| PDL1 | FACS | Biolegend | Clone 29E.2A3 |
| HLA-A2 | FACS | Biolegend | Clone BB7.2 |
| CD25 | FACS | BD | Clone M-A251 |
| CD137 | FACS | Biolegend | Clone 4B4-1 |

**Supplementary Table 1:** Details of the antibodies utilised in this study.
