# Supplementary material for: Deletion of the deISGylating enzyme USP18 enhances tumour cell antigenicity and radiosensitivity
Source: Br J Cancer. 2020 Nov 20;124(4):817–30. doi: 10.1038/s41416-020-01167-y (PMC7884788; doi:10.1038/s41416-020-01167-y)
Supplement: Supplementary file 2 — Supplementary Data [file 41416_2020_1167_MOESM2_ESM.docx]

**Supplementary Information**

**Deletion of the deISGylating enzyme USP18 enhances tumour cell antigenicity and radiosensitivity**

***Adan Pinto-Fernandez^1,2,8^; Mariolina Salio^3^; Tom Partridge^4^; Jianzhou Chen^5^; George Vere^1^; Helene Greenwood^1^; Cyriel Sebastiaan Olie^1^; Andreas Damianou^1^; Hannah Claire Scott^1^; Henry Jack Pegg^6^; Alessandra Chiarenza^6^; Laura Díaz-Saez^1,7^; Paul Smith^1,7^; Claudia Gonzalez-Lopez^3^; Bhavisha Patel^6^; Emma Anderton^6^; Neil Jones^6^; Tim R. Hammonds^6^; Kilian Huber^1,7^; Ruth Muschel^4^; Persephone Borrow^5^; Vincenzo Cerundolo^3^ and Benedikt M. Kessler^1,2,8^***

*^1^TDI Mass Spectrometry Laboratory, Target Discovery Institute, Nuffield Department of Medicine, University of Oxford, Oxford, Roosevelt Drive, Oxford OX3 7FZ, UK*

*^2^Chinese Academy of Medical Sciences Oxford Institute, Nuffield Department of Medicine, University of Oxford, Oxford, Roosevelt Drive, Oxford OX3 7FZ, UK*

*^3^MRC Human Immunology Unit, MRC Weatherall Institute of Molecular Medicine, Radcliffe Department of Medicine, University of Oxford, Oxford OX3 9DS, UK*

*^4^Nuffield Department of Clinical Medicine, University of Oxford, Roosevelt Drive, Oxford OX3 7FZ, UK*

*^5^CRUK/MRC Oxford Institute for Radiation Oncology, Department of Oncology, University of Oxford, Oxford, OX3 7DQ, UK*

*^6^CRUK Therapeutic Discovery Laboratories, London Bioscience Innovation Centre, London, NW1 0NH, UK*

*^7^Centre for Medicines Discovery, Nuffield Department of Medicine, University of Oxford, Oxford, Roosevelt Drive, Oxford OX3 7FZ, UK*

**Running Title:** New roles for USP18 in cancer therapy

- ^8^**Corresponding authors:**

Adan Pinto-Fernandez

Target Discovery Institute

Nuffield Department of Medicine

University of Oxford

Oxford OX3 7FZ, UK

Email: adan.pintofernandez@ndm.ox.ac.uk

Benedikt M Kessler

Target Discovery Institute

Nuffield Department of Medicine

University of Oxford

Oxford OX3 7FZ, UK

Email: benedikt.kessler@ndm.ox.ac.uk


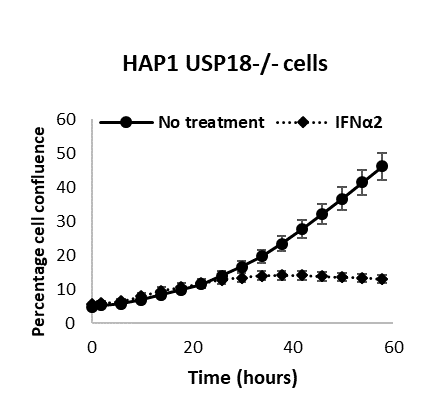

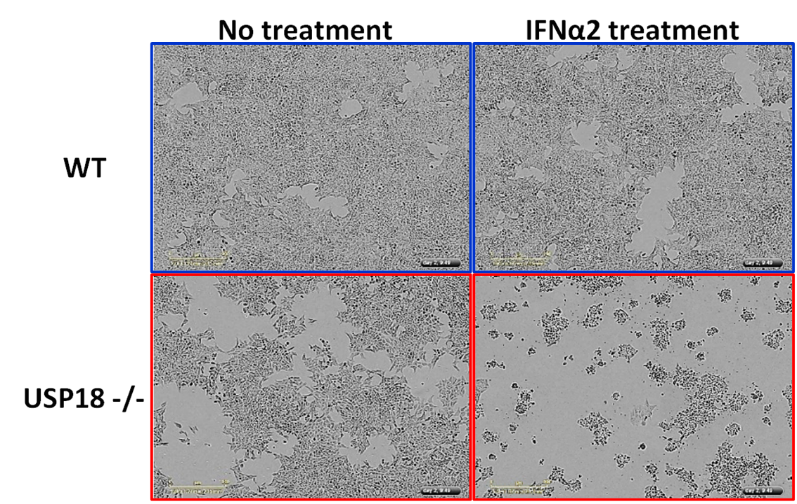


**C**

**B**

**A**

**D**


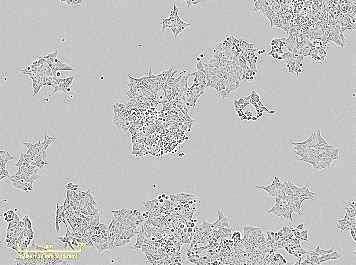

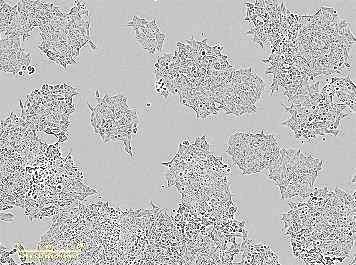

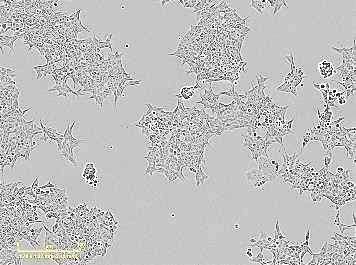

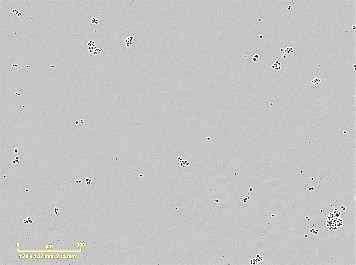

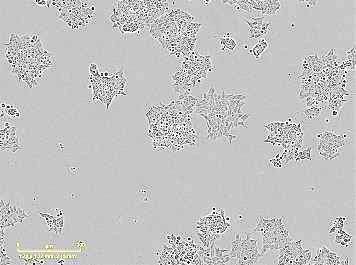

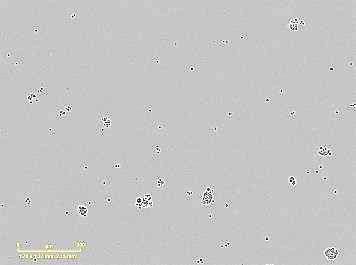


**EV**

**WT**

**C64R/C65R**

**IFN**

**(82h.)**

**NT**


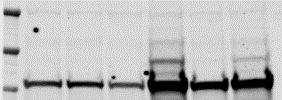

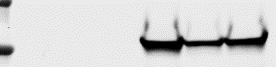

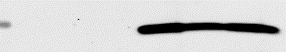


**75-**

**100-**

**150-**

**250-**

**50-**

**15-**

**-ISG15**

**-IFIT3**

**-HERC5**

**-HERC5-ISG15**

**IFN (32h.) : - - - + + +**

**EV**

**USP18 wt**

**C64R/C65R**

**EV**

**USP18 wt**

**C64R/C65R**


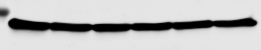


**- GAPDH**

**37-**

**E**


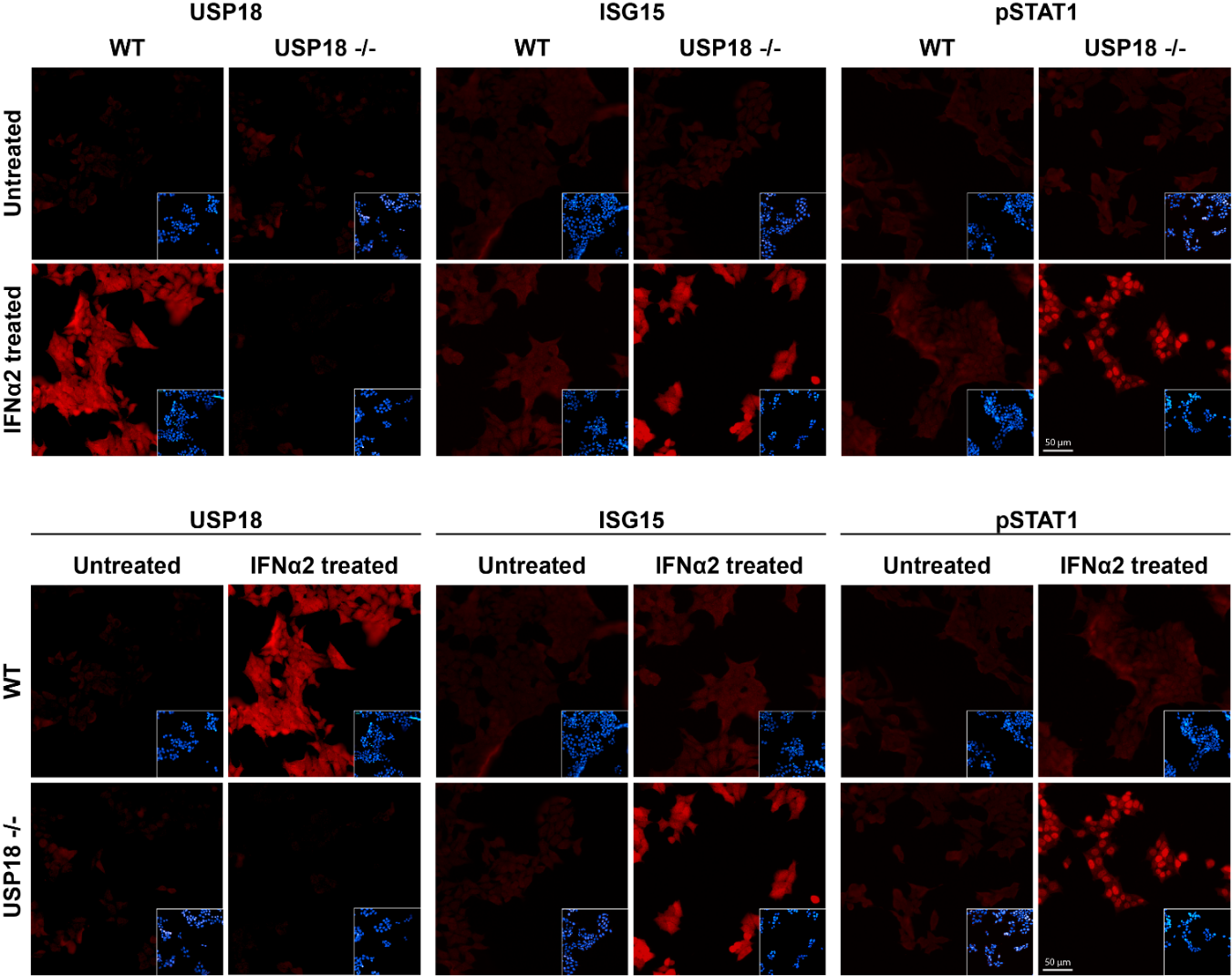


**E**

**D**


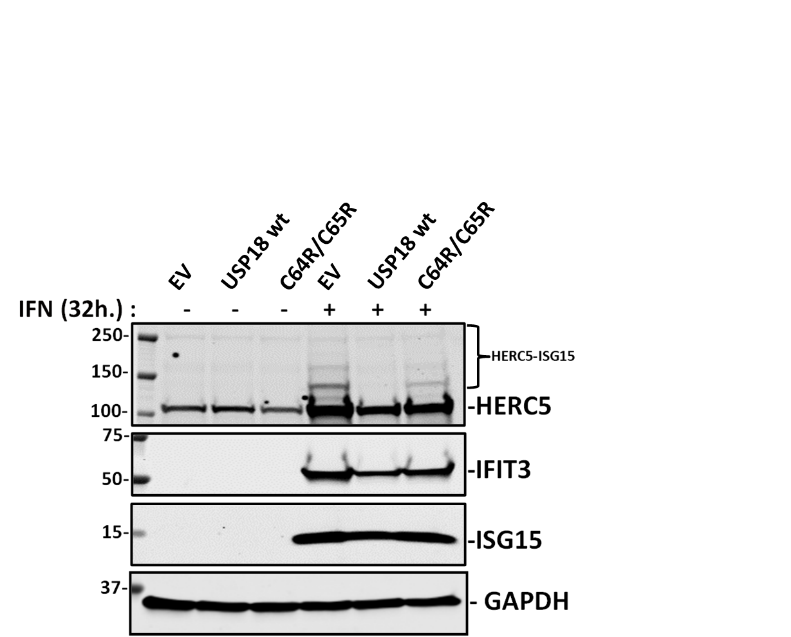

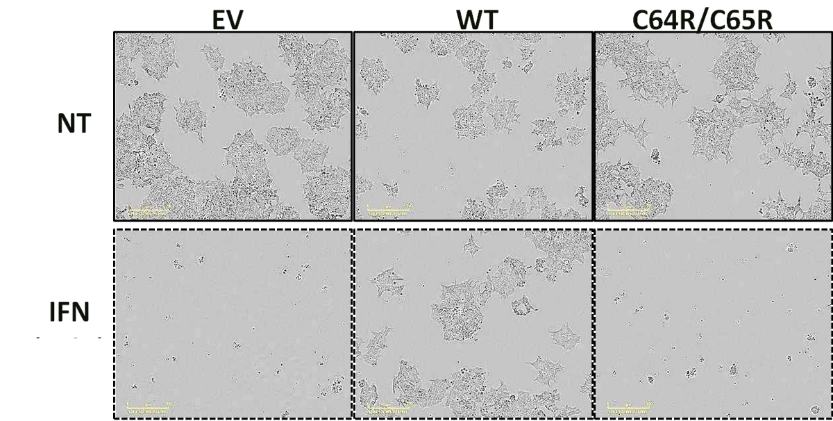


**Figure S1: USP18 alters protein ISGylation and cell viability in the presence of IFN.**  **A)** USP18-deficient HAP1 cell confluence (IncuCyte data) in the presence/absence of IFN. **B)** Cell confluence and morphology of WT and USP18 KO cells after 58 hours of incubation with IFN (phase contrast images). **C)** Confocal microscopy immunofluorescence images displaying a strong accumulation of USP18 in WT cells, ISG15 and phosphorylated STAT1 (pSTAT1) in the USP18-deficient cells after 24 hours treatment with IFN. **D)** Cell confluence and morphology analysis showing that stable re-expression of USP18 WT, but not of a catalytically inactive mutant USP18 C64R/C65R, in USP18-deficient cells rescue them from the cytotoxic effects of IFN (82 hours treatment; EV: empty vector; NT: no treatment). **E)** Immunoblot of cell extracts from USP18 KO cells expressing USP18 WT and USP18 C64R/C65R. Ectopic expression of WT, and in a lesser extent catalytically inactive USP18, prevents IFN signalling as the expression of the ISGs HERC5, IFIT3 and ISG15 is reduced in these conditions. GAPDH was used as a loading control in the immunoblots (same experiment as in Figure 1G). Supporting data for Fig. 1.

**
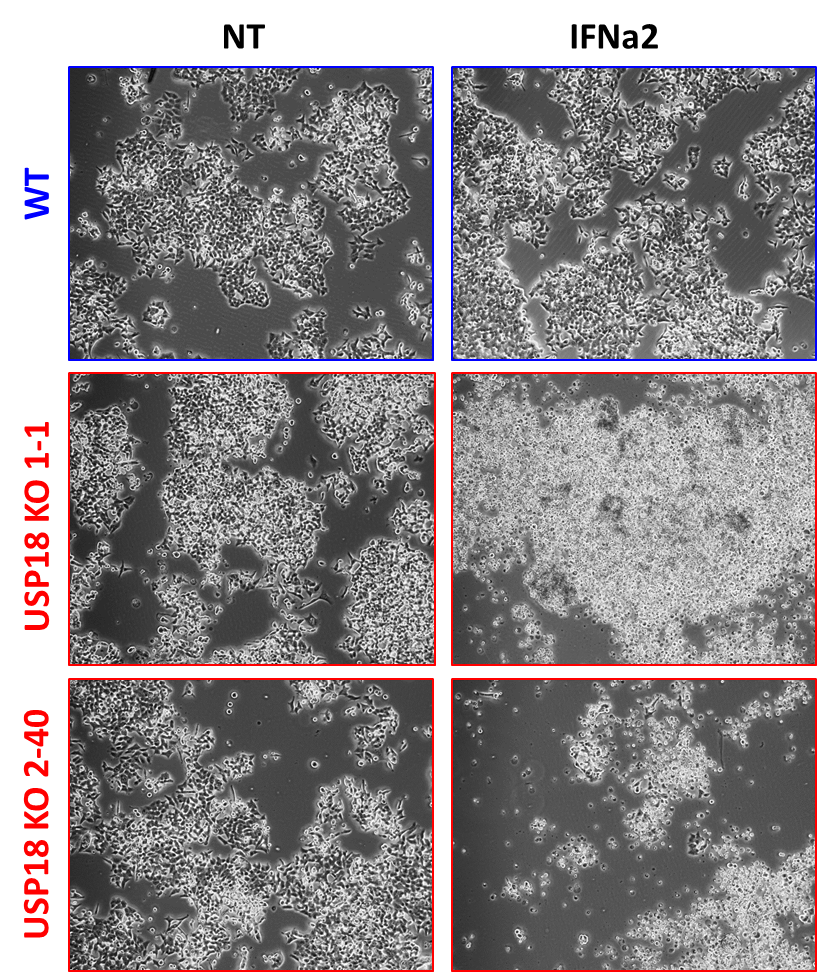
**

**B**

**A**

**Cell Viability (72 h.)**

**D**

**C**

**
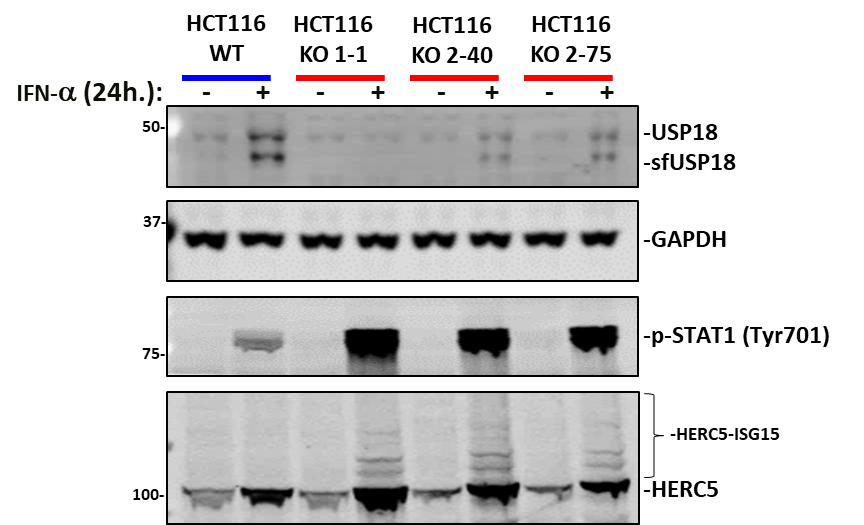

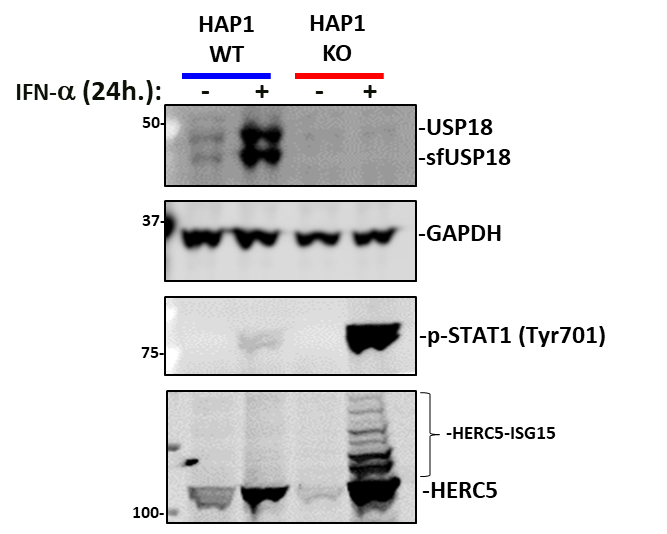
**

**Figure S2: USP18 alters protein ISGylation and cell viability in the presence of IFN in HCT116 colorectal carcinoma cells. A)** Cell viability after 72 hours (resazurin assay) in three different clones of USP18-deficient (1-1, 2-40, and 2-75) and and parental wild type HCT116 cells, and HAP1 cells in the presence/absence of IFN. **B)** Cell confluence and morphology of WT and USP18 KO cells after 72 hours of incubation with IFN (phase contrast images). **C)** Immunoblot of cell extracts from USP18 KO and wild type cells expressing after 24 hours of treatment with and without IFN. **D)** Same conditions as in C) but using HAP1 cells, further supporting our results showing, enhanced activation of the pathway (shown as increased phosphorylation of STAT1) and ISGylation of HERC5 in the USP18-defficitent cells, after IFN treatment.

**
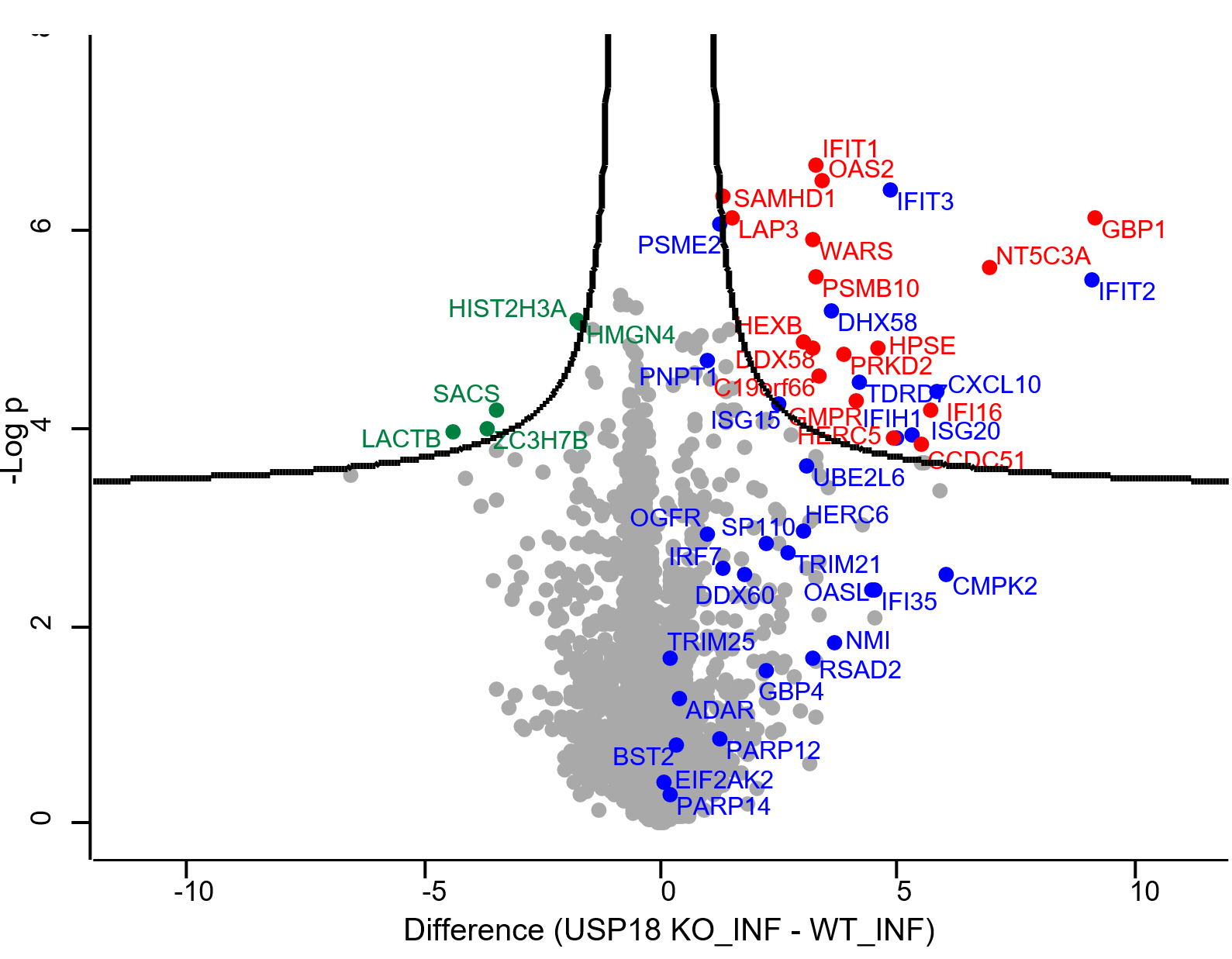
**


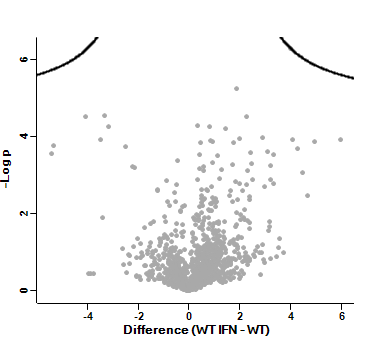


**-Log p (FDR: 0.01; s0: 0.1)**

***HAP1 wt GlyGly peptides***

***USP18-dependent proteome (with IFN)***

**B**

**A**

**
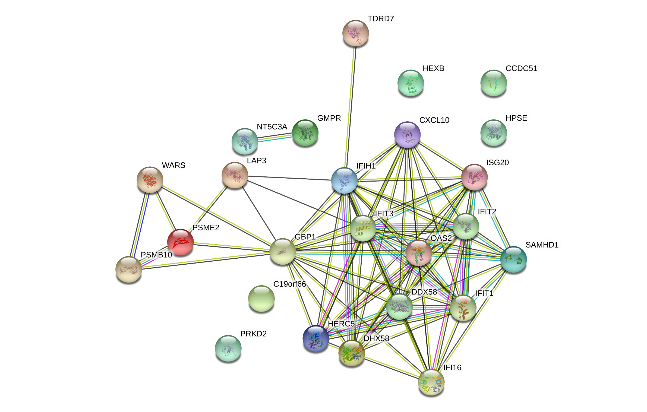

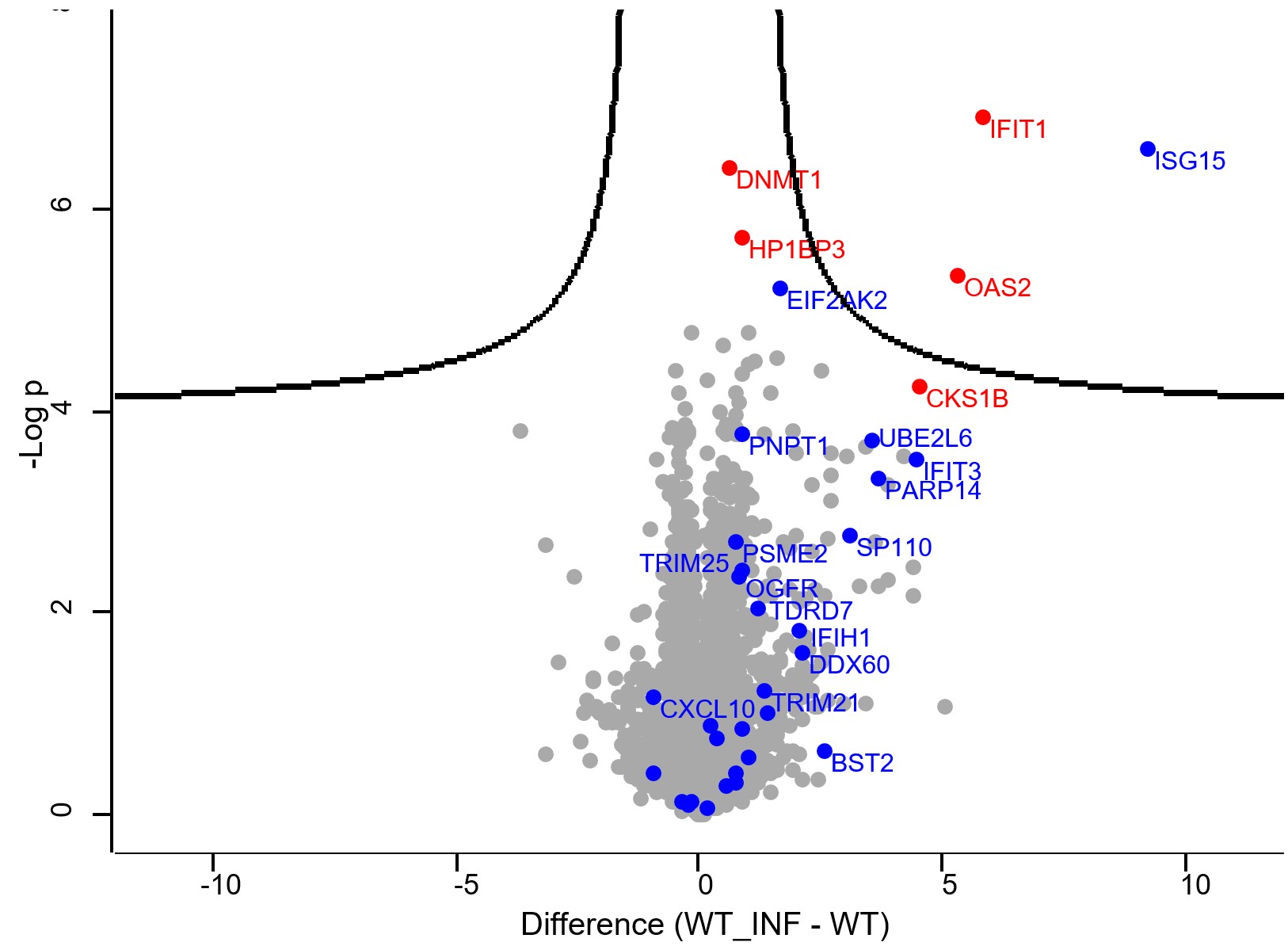
**

**E**

**D**

***USP18-dependent proteome***

***IFN-dependent proteome***

**C**

**
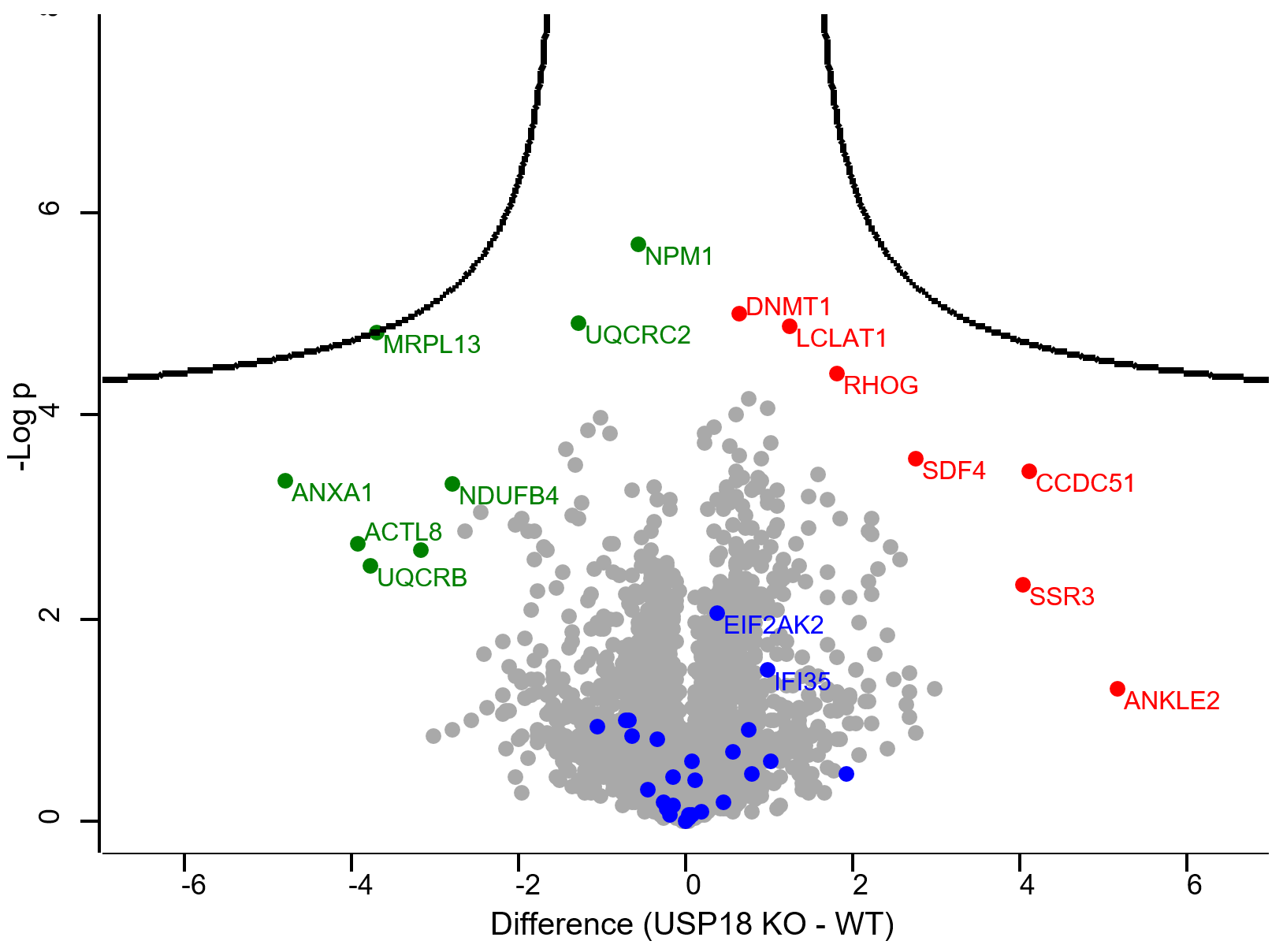
**


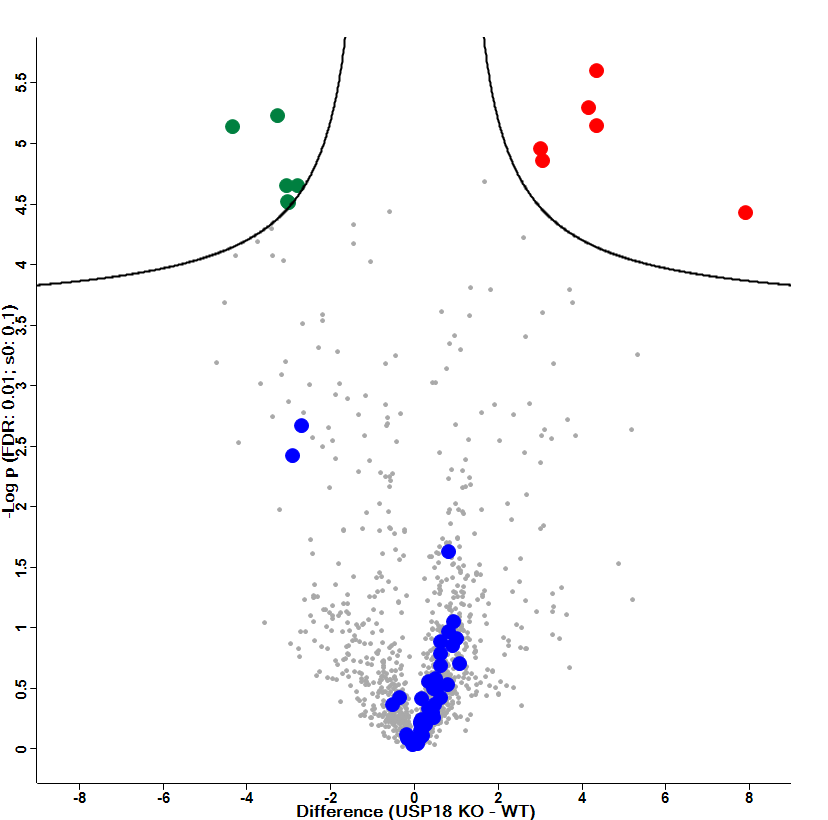


**ZPR1**

**MLLT1**

**SPG20**

**TKT**

**HNRNPU**

**H2AFV**

**IGF1R**

**SCAMP3**

**EEF2**

**HIST1H1E**

**MIEF1**

**RPL6**

***HAP1 USP18 KO vs wt GlyGly peptides***

**F**

**G**

**
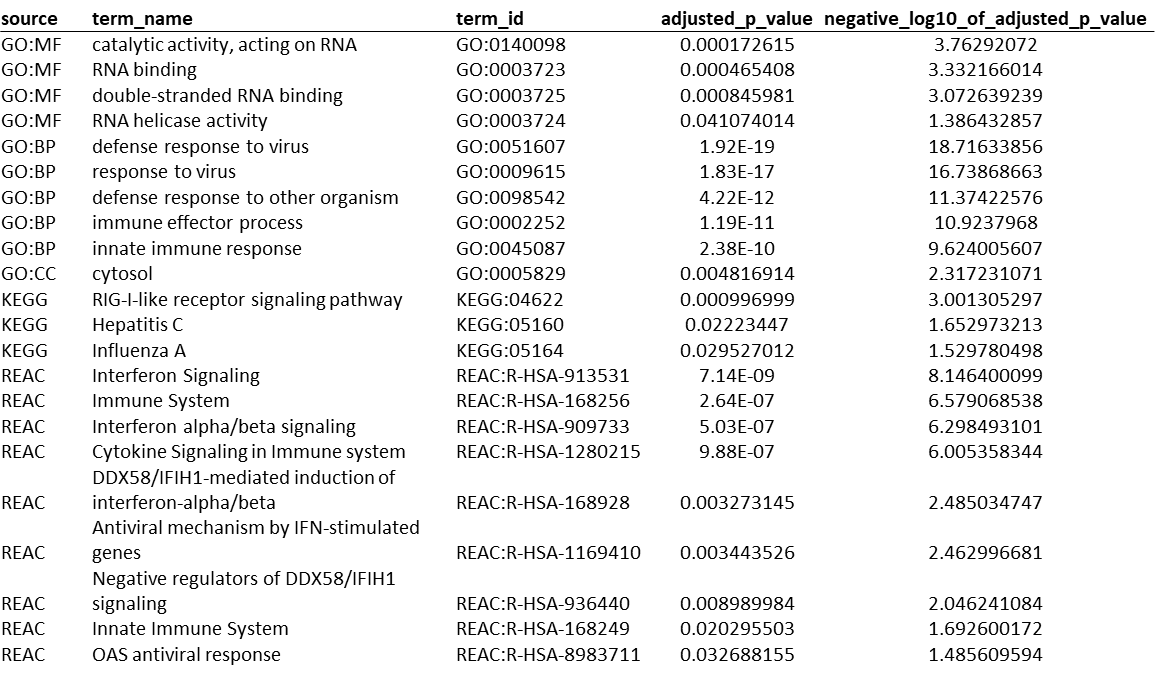
**

**Figure S3: Analysis of the USP18-dependent proteome. A)** Comparative volcano plot of the GlyGly modified peptides in HAP1 WT cells treated with IFN for 48 h. and untreated WT. No significant changes were observed using the statistical cut-off values used for all the proteomic analyses performed in this study (FDR: 0.01; s0: 0.1). Volcano plots showing the differential expression of proteins in the following comparisons: USP18 KO plus IFN vs WT plus IFN **B)**, USO18 KO vs WT **C)**, WT plus IFN vs WT **D)** (statistical parameters: FDR = 0.01; So = 0.1). STRING functional protein association networks analysis **E)** and g:PROFILER pathway enrichment analysis **F)** of the proteins signifantly up-regulated in A). **G)** Comparative volcano plot of the GlyGly modified peptides in HAP1 USP18 KO cells and WT cells (untreated).


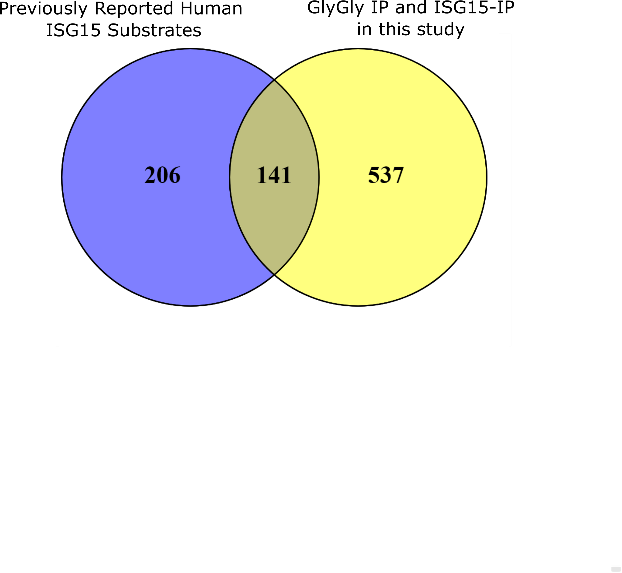


**B**

**A**

**
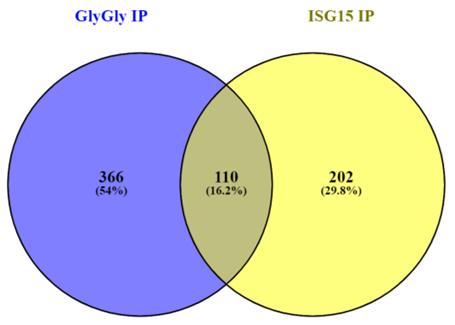
**

**Figure S4: USP18-deletion exacerbates the cellular ISGylated protein network. A)** Venn diagram showing the overlap between the GlyGly peptide IP and the ISG15 IP, performed in the same experimental conditions (HAP1 USP18 KO with IFN vs. HAP1 WT with IFN). **B)** Venn diagram comparing the depth of this ISGylome study and previously reported ISGylomes (this data can be found in Supplementary Data Table 4). Supporting data for Fig. 3.

**Supplementary Table 2:** Comparative between the up-regulated proteins in melanoma patients responding to anti-PD1 therapy ^50^ and our different USP18-dependent (USP18 KO cell plus IFN vs WT plus IFN) proteomic analysis datasets (Full proteome, GlyGly ISGylome and ISG15 interactome). (UP = up-regulated, DOWN = down-regulated, (s) = significant (t-test with permutation FDR = 0.01 for multiple-test correction and s0 = 0.1 as cut-off parameters) and (ns) = no significant.
